# The Revolution of Immunotherapy in Gynecological Cancers: The Lazarus Effect in Endometrial Cancer

**DOI:** 10.3390/jcm12175540

**Published:** 2023-08-25

**Authors:** Monika Ducceschi, Maggie Polignano, Marta Bini, Salvatore Lopez, Elena Conca, Elena Tamborini, Federica Perrone, Giulia Carlo Stella, Maria Cristina Petrella, Rosaria Carciotto, Grazia Artioli, Valeria Maffeis, Lucia Sartor, Francesco Raspagliesi, Mara Mantiero

**Affiliations:** 1 Gynecological Oncology Unit, Fondazione IRCCS Istituto Nazionale dei Tumori di Milano, 20133 Milan, Italy; monika.ducceschi@istitutotumori.mi.it (M.D.); maggie.polignano@istitutotumori.mi.it (M.P.); marta.bini@istitutotumori.mi.it (M.B.); salvatore.lopez@istitutotumori.mi.it (S.L.); francesco.raspagliesi@istitutotumori.mi.it (F.R.); 2 Department of Innovation Diagnostics, Laboratory of Molecular Pathology, Fondazione IRCCS Istituto Nazionale dei Tumori di Milano, 20133 Milan, Italyelena.tamborini@istitutotumori.mi.it (E.T.); federica.perrone@istitutotumori.mi.it (F.P.); 3 Department of Hematology, Oncology, and Molecular Medicine, Grande Ospedale Metropolitano Niguarda, 20162 Milan, Italy; giulia.carlostella@ospedaleniguarda.it; 4 Oncologia Medica Ginecologica, Azienda Ospedaliera Universitaria Careggi, 50134 Firenze, Italy; mariacristina.petrella@gmail.com; 5 Oncologia Medica, Ospedale Maria Paternò Arezzo, 97100 Ragusa, Italy; rosaria.carciotto@asp.rg.it; 6 Oncologia Medica, Ospedale Cà Foncello, 31100 Treviso, Italy; grazia.artioli@aulss2.veneto.it; 7 Anatomia Patologica, Ospedale Cà Foncello, 31100 Treviso, Italy; valeria.maffeis@aulss2.veneto.it; 8 Oncologia Medica, Ospedale Camposampiero e Cittadella (Padova) ULSS 6, 35012 Euganea, Italy; lucia.sartor@aulss6.veneto.it

**Keywords:** Dostarlimab, endometrial cancer, cancer immunotherapy, immune checkpoint molecule, immune checkpoint blockade, Lazarus effect, Lazarus response

## Abstract

Patients with solid tumors and mismatch repair deficiency (dMMR) or microsatellite instability-high (MSI-H) are eligible for immunotherapy. Recently, different reports described patients with poor performance status (PS), unrelated to comorbidities, which showed a rapid improvement of their clinical conditions under immunotherapy, which evoked a Lazarus response. Very few data on the efficacy and safety of immunotherapy in patients with gynecological malignancies and poor PS are available. Based on the GARNET trial, Dostarlimab, a monoclonal antibody anti-programmed death receptor-1 (PD-1), has been approved in advanced or recurrent mismatch repair deficient endometrial cancer (EC) which progressed after platinum-based therapy. For the first time, in gynecological oncology, an immune checkpoint inhibitor drastically changed the clinical practice. We collected a multicenter case series of six patients with advanced endometrial carcinoma and PS ECOG 3–4 treated with Dostarlimab, showing exceptionally quick responses and significant improvement of PS to configure a Lazarus response.

## 1. Introduction

Endometrial cancer is the most common gynecologic tumor in developed countries [1]. Risk factors for EC are well known and are strongly correlated with lifestyle factors (e.g., obesity and diabetes), increasing age and socioeconomic-driven changes as parity [2]. Most patients are diagnosed in the early-stage of disease and have a good prognosis. However, in advanced-stage, the five-year survival rate falls to 18% [3]. For a long time, Carboplatin and Paclitaxel-based chemotherapy has been the gold standard for advanced disease with unsatisfactory results: nearly half of the patients have disease progression within a year.

In 2013, TCGA (The Cancer Genome Atlas) proposed a new classification of EC which is based on molecular profiling [4]. Molecular subgroups correlate closely with disease prognosis; therefore, there is a shift combining molecular and histological classifications of EC [5]. The new TCGA approach especially highlighted an interesting subgroup of EC (about 30%) with mismatch repair deficient (dMMR) or microsatellite instability-high (MSI-H) and intermediate prognosis. The loss of expression of one or more of the MMR proteins (MLH1, MSH2, MSH6, and PMS2) can be detected by immunohistochemistry and MSI-H can be evaluated via Polymerase Chain Reaction (PCR). Testing EC for MMR/MSI has diagnostic (screening for Lynch syndrome), prognostic and predictive (immune checkpoint inhibitors) value. In 2017, the Food and Drug Administration (FDA) approved immune checkpoint inhibitor (ICIs) Pembrolizumab for the treatment of dMMR/MSI-H solid tumors, including EC. Pembrolizumab became the first biomarker-based and agnostic systemic therapy for cancer [6]. Moreover, in 2021, based on the GARNET trial, the FDA granted accelerated approval for Dostarlimab monotherapy, for patients with dMMR advanced or recurrent EC, which progressed after platinum-based therapy [7]. These were the first steps of a new era in oncology, opening the doors to new drugs with important changes in real life practice: patients with advanced cancers who previously had limited treatment options may now benefit from immunotherapies that offer long-lasting responses and improved survival outcomes. In EC, especially, the phase 3 RUBY trial (ENGOT-EN6-NSGO/GOG3031) showed that adding immunotherapy (Dostarlimab) to standard chemotherapy in the first-line setting significantly improves progression-free survival (PFS) compared to chemotherapy alone, with a promising early sign of improved overall survival (OS) becoming the new standard of care [8].

In oncology, the Lazarus effect is a phenomenon described, for the first time, with the introduction of targeted therapies for patients with oncogene-addicted cancers. It is characterized by extraordinary activity of these drugs in terminally ill patients with poor performance status (PS) and critically high disease burden, with rapid improvement of patients’ health conditions during the very first days of treatment. The advent of such “Lazarus responses” led to a revolution in the treatment decision making for patients who were otherwise candidates only for best supportive care. The presence of predictive biomarkers associated with the high chance of treatment response enables their access to target therapies [9].

In this new context, emerging scientific data reported excellent responses with salvage immunotherapy in patients with dMMR/MSI-H metastatic solid tumors in end-stage disease. ICIs or target therapy can induce potentially long-lasting “Lazarus responses”. However, differently to melanoma or lung cancer, no data are available regarding the “Lazarus effect” in gynecological tumors, due to its recent approval.

Here we report an Italian case series of patients with advanced EC and extremely poor PS related to high disease burden, treated with Dostarlimab, showing exceptional and quick responses with significant improvement of PS to configure a Lazarus response.

## 2. Methods

**Figure 1:**
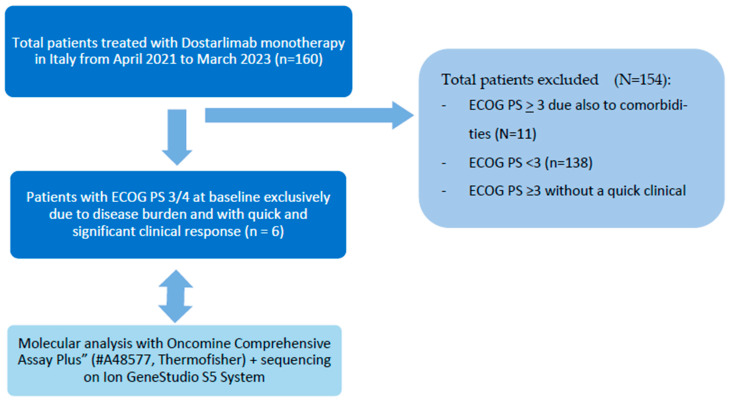
Patients’ enrollment.

We collected data of patients affected by recurrent or metastatic MSI-H/dMRR EC treated with Dostarlimab monotherapy in Italy from April 2021 to March 2023. Among them, we selected patients with ECOG PS 3–4 at baseline, exclusively due to disease burden, and who experienced a significant and quick clinical response. We identified 6 patients with these characteristics treated in 6 different Oncologic Institutes in Italy (Figure 1). Dostarlimab 500 mg was administered with endovenous infusion over 30 min, every 3 weeks for 4 times; then 1000 mg every 6 weeks. Administration of Dostarlimab was continued, according to the recommended schedule, until disease progression or unacceptable toxicity.

We retrospectively collected the formalin-fixed and paraffin-embedded (FFPE) specimens of EC of all six patients. The neoplastic tissue was analyzed using targeted-DNA panel “Oncomine Comprehensive Assay Plus” (#A48577, Thermofisher (Waltham, MA, USA)), and sequenced on the Ion GeneStudio S5 System leveraging the Ion Torrent platform.

Descriptive statistics were used to summarize the observed events.

### Case Series

**Case 1:**

A 54-year-old woman presented with a 5-year history of endometrial cancer. She received the first diagnosis in 2016 when she underwent hysterectomy with annexectomy and pelvic lymphadenectomy for a FIGO stage IB endometrioid endometrial carcinoma and adjuvant brachytherapy. In May 2021, the appearance of deep vein thrombosis extended to the right leg and thigh allowed the discovery of a disease relapse. Revision of the histological exam in tertiary cancer care confirmed the above-mentioned diagnosis and found loss of nuclear expression of MLH1 and PMS2 in tumor cells.

Two cycles of first-line chemotherapy with Carboplatin AUC5 and Paclitaxel 175 mg/mq every 3 weeks were administered until August 2021. The patient had a rapid decline of the clinical conditions with lumbar and bilateral legs pain that prevented her from walking or sitting, leading to a PS ECOG 4. She was urgently transferred in our clinic and the radiologic assessment demonstrated bone lesions, especially at the lumbar and pelvic level.

**Figure 2:**
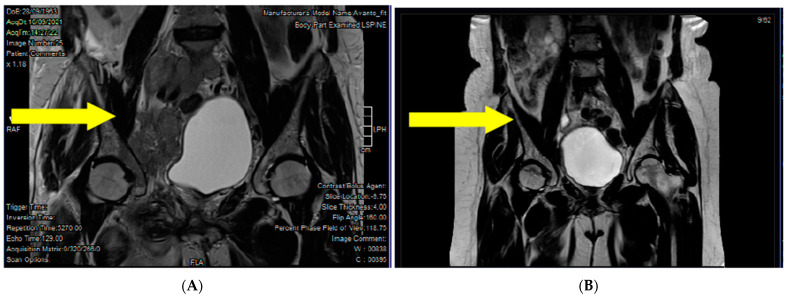
Radiologic response to Dostarlimab at (**A**) baseline (September 2021) and (**B**) after 4 weeks of treatment (October 2021). Yellow arrow points towards the lesions.

The patient received palliative radiotherapy on the spine, started zoledronic acid for lytic bone lesions, and thanks to the microsatellite instability, she had access to treatment with Dostarlimab. After a few weeks of therapy, the patient was hardly recognizable. She came by herself in the outpatient clinic; she could start to walk by herself and eliminated all the painkillers. The radiological assessment performed after 4 weeks demonstrated a response of >50% of all the known lesions (Figure 2). She is actually a PS ECOG 0 and is continuing treatment after 20 cycles.

**Case 2:**

In June 2018, a 59-year-old woman underwent hysterectomy, bilateral annexectomy, and sentinel lymph node, with diagnosis of endometrial carcinoma endometrioid type. The disease was very localized, FIGO Stage IA, so the patient did not receive any adjuvant treatment.

In May 2020, the patient presented with a deep vein thrombosis of the left leg, which allowed the discovery of a massive disease relapse with a ubiquitous lymph node involvement and a pelvic mass. A new histological assessment was performed which confirmed the known endometrial neoplasia and molecular analysis demonstrated methylation of the promoter of *MLH1*. The patient underwent first-line of chemotherapy with Carboplatin area under the curve (AUC) 5 and Paclitaxel 175 mg/mq every three weeks. After 3 cycles of therapy, the patient presented a clinical disease progression with difficulty in walking due to worsening of the left leg (very edematous and hard), appearance of intense asthenia and of vomiting with difficulty in food intake, defining an ECOG PS 3. Radiological assessment confirmed the disease progression both of the deep vein thrombosis, actually involving the entire leg up to the groin, and an important dimensional increase of the known lymphadenopathies. Supportive therapy with opioids and parenteral nutrition was initiated.

**Figure 3:**
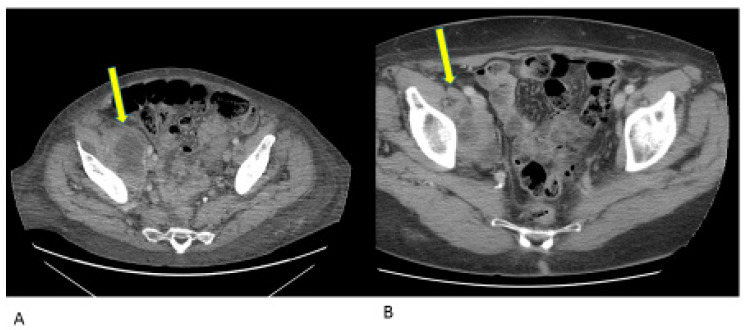
Radiologic best response to Dostarlimab at (**A**) baseline (December 2020) and (**B**) after 10 months of treatment (October 2021). Yellow arrow points towards the lesions.

In January 2021, thanks to the microsatellite instability and via the nominal use access, the patient started Dostarlimab in monotherapy. A striking clinical response was observed only after one cycle of therapy. The patient could start to feed by herself and was weaned of opioids with a ECOG PS 0. The first radiologic evaluation demonstrated a significant dimensional response at the lymphnodal level, whereas the pelvic mass increased, due to necrosis. The patient thus continued treatment until February 2022 when she had a lymph nodal progression for which she underwent palliative radiotherapy (Figure 3).

**Case 3:**

A 62-year-old woman was referred for a pelvic relapse of endometrial carcinoma. Her story begins in 2019 when she underwent a hysterectomy with bilateral annexectomy and sentinel lymph node biopsy for an endometrioid type endometrial carcinoma FIGO stage IB, with a p53 mutant pattern and loss of MLH2 by immunohistochemistry. Due to the characteristics of the disease, the patient was eligible for adjuvant chemotherapy and radiotherapy. The patient ended pelvic radiotherapy (50 Gy) in March 2020 and refused systemic treatment. A genetic counseling was performed excluding Lynch Syndrome.

In October 2020, the CT scan demonstrated a pathological lombo aortic lymphadenopathy, which was treated with Cyberknife radiotherapy.

In May 2021, the patient presented another relapse with a pelvic mass infiltrating the rectum, for which she received a first-line of chemotherapy with Carboplatin AUC5 and Paclitaxel 175 mg/mq followed by maintenance treatment with aromatase inhibitors.

In January 2022, due to lumbar pain, the patient performed a radiological assessment showing pathological tissue englobing the right ureter conditioning hydroureteronephrosis, pelvic carcinosis and pathological overclave and tracheal lymph nodes. Clinical conditions rapidly deteriorated to ECOG PS 3 and painkillers were started according to the patient’s symptoms and a right nephrostomy was placed.

**Figure 4:**
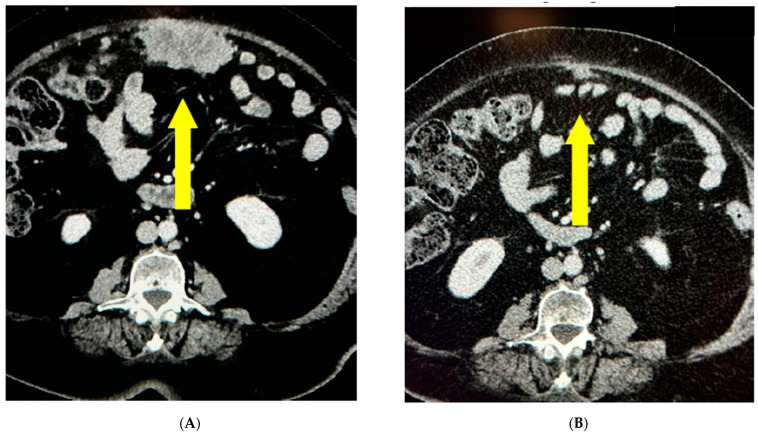
Radiologic response to Dostarlimab at (**A**) baseline (January 2022) and (**B**) after 3 cycles of treatment (April 2022). Yellow arrow points towards the lesions.

Dostarlimab was started in February 2022, and after 2 cycles, the clinical benefit was very important, such as painkillers being stopped. CT scan demonstrated a partial response of disease and a ureteral stent was positioned and nephrostomy removed (Figure 4). This resulted in a very good quality of life for the patient, who continued to work and travel thanks to the rapid improvement of ECOG PS (PS 1).

Despite the clinical and radiological benefit demonstrated, the patient presented a pelvic mass in December 2022 leading to treatment discontinuation. The patient has now started a third line of treatment with Carboplatin and pegylated liposomal doxorubicin (PLD), which is still ongoing.

**Case 4:**

**Figure 5:**
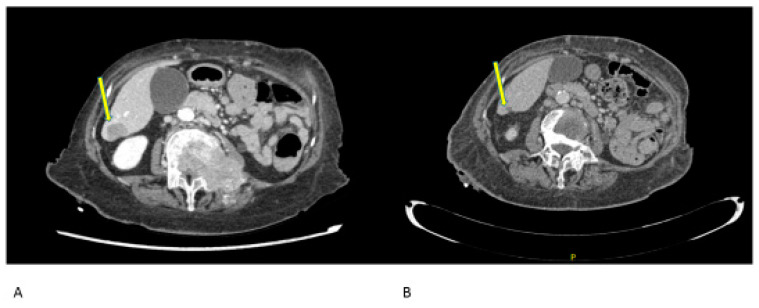
Radiologic evaluation of hepatic lesions and L3 lesion with involvement of the medullary canal during Dostarlimab treatment at baseline (November 2021), 2 and 7 cm, respectively, figure (**A**) and after 4 cycles of immunotherapy (February 2022), 6 mm and no more visible, the paravertebral lesion of L3, figure (**B**). Yellow arrow points towards the lesions.

A 73-year-old woman presented with a two-year history of metastatic endometrial cancer. In March 2021, the patient was diagnosed with endometrioid adenocarcinoma of the endometrium, stage IV due to lung and bone metastases. A comprehensive genome sequencing panel demonstrated the presence of microsatellite instability and a high tumor mutational burden (37.83 mutations-per-megabase). The patient was considered eligible for first-line chemotherapy with Carboplatin AUC 5 and Paclitaxel 175 mg/mq every 3 weeks for 6 cycles. The CT scan performed demonstrated hepatic, pelvic, lung and bone disease progression. The clinical conditions rapidly deteriorated, partly due to the bone disease conditioning, a vertebral fracture for which the patient started opioids, and in part due to the pelvic disease leading to vaginal bleeding, for which she needed fibrinolytic therapy and blood transfusions. A palliative radiotherapy for antalgic purpose was performed on the vertebral fracture and at haemostatic goal on the vaginal lesion. Due to the frailty of clinical conditions, PS ECOG 3, the patient was hospitalized during radiotherapy as she was not able to support outpatient cures and was, thus, not considered eligible for another chemotherapy. In light of the microsatellite instability, Dostarlimab was started in November 2021. After 3 cycles of therapy, the patient had a resounding clinical response, leading to a ECOG PS 0: she was able to autonomously walk around and come to the outpatient treatments. The first radiological evaluation, performed after 4 cycles, showed a partial response in all lesions (Figure 5). The patient was weaned off painkillers and is regularly practicing physiotherapy at home. The last CT scan, performed in February 2023, confirmed the disease response already seen in previous radiologic evaluations, and the treatment is still ongoing.

**Case 5:**

A 61-year-old woman received in October 2020 a radical surgery (hysterectomy, bilateral annexectomy, pelvic lymphadenectomy and omentectomy) for a FIGO stage IIIC mixed endometrial cancer composed of high grade serous carcinoma (60%) and endometrioid carcinoma (40%).

She underwent adjuvant treatment according to the sandwich strategy, including systemic chemotherapy with Carboplatin AUC 5 and Paclitaxel 175 mg/mq every three weeks and pelvic radiotherapy, concluded in July 2021.

**Figure 6:**
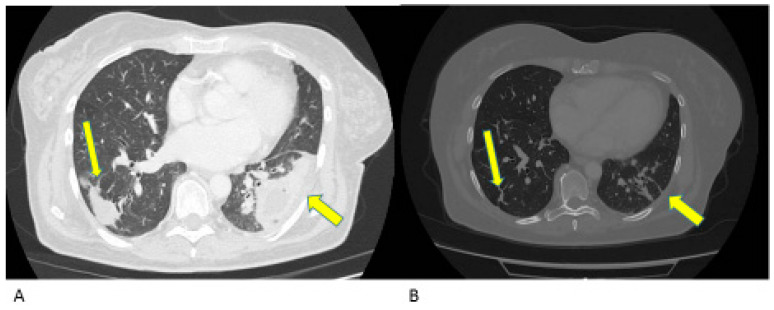
Pulmonary response during Dostarlimab treatment at (**A**) baseline (June 2022) and (**B**) May 2023. Yellow arrow points towards the lesions.

The disease was platinum-resistant and the radiological evaluation of October 2021 demonstrated a pelvic and pulmonary relapse, for which the patient started a monochemotherapy with PLD. In a few months, the clinical conditions dramatically declined. The patient was PS ECOG 3. She needed a wheelchair; as soon as she would get out of her house, abdominal pain and cough appeared, so opioids were started; and she had anemia, needing blood transfusions support. The CT scan demonstrated a radiological lymphnodal and pulmonary progression (Figure 6). A molecular analysis was performed, showing a microsatellite instability.

Thus, the patient was able to start Dostarlimab monotherapy in March 2022. After 2 cycles of therapy, the patient did not need painkillers anymore, the cough disappeared, the hemoglobin went back to normal values, and she started to walk again and went back to her work place. She is still on treatment after 16 months with an ECOG PS 1.

**Case 6:**

A 75-year-old woman was referred in March 2022 due to the appearance of deep vein thrombosis. She had a history of breast cancer treated with surgery, radiotherapy and hormonal treatment, and of an endometrioid endometrial carcinoma FIGO stage II, for which she received radical surgery. During hospitalization, she underwent a CT scan which showed lung and abdominal nodules. A histological assessment was performed, demonstrating a gynecological origin of the relapse. The molecular analysis also showed a loss of nuclear expression of MLH1 and PMS2 in neoplastic cells.

On April 2022, the patient started a first-line standard chemotherapy with Carboplatin AUC 5 and Paclitaxel 175 mg/mq every three weeks. After 6 cycles, the patient started to have dyspnea and cough, abdominal pain with difficulty in food intake and lumbar pain. Supportive therapy with painkillers and nutritional support was started. The patient also received palliative radiotherapy at the lumbar level.

**Figure 7:**
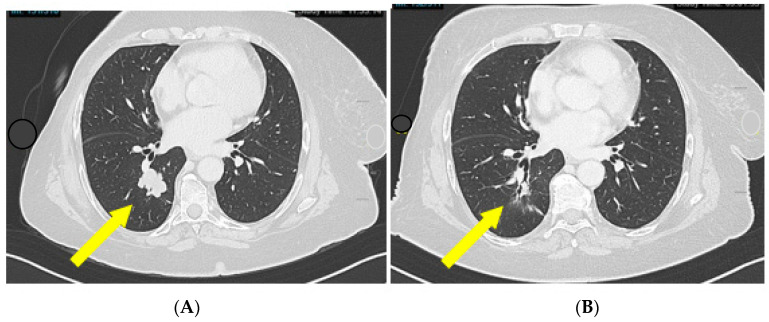
Pulmonary response during Dostarlimab treatment at (**A**) baseline (October 2022) and (**B**) after 6 cycles. Yellow arrow points towards the lesions.

The patient started Dostarlimab monotherapy on October 2022 with an ECOG PS 3. After two cycles of treatment, the patient had an important clinical benefit with the disappearance of pain and gradual recovery of food intake (Figure 7). The radiological assessment demonstrated a stable disease at the first CT scan performed at 12 weeks; however, from the second radiologic evaluation, a partial response was evident. The patient, after 8 months, is thus, still receiving treatment and she is actually an ECOG PS 0.

## 3. Results

The median age of patients was 65 years (range 54–75). Four patients presented with early FIGO stage (I–II) at diagnosis. Five patients received just one previous line of chemotherapy and started Dostarlimab 500 mg every 21 days after progression to Carboplatin and Paclitaxel.

All patients started immunotherapy with a PS ECOG ≥ 3, which was exclusively due to disease burden. Patients presented various symptoms, including uncontrolled pain despite analgesic therapy, difficulty in food intake, cough and also anemia necessitating blood transfusions. The median time of clinical response (from PS ECOG 3–4 to PS ECOG 0–1) was 6 weeks (range 3–9) (Table 1). All patients had a complete resolution of symptoms within this time and could come by themselves in the outpatient clinic. All patients at the first radiological evaluation (performed within 12 weeks from cycle 1) showed an important dimensional reduction of disease burden. A complete response was reached in two patients.

## 4. Molecular Results

Concerning molecular analysis, among the initial six patients, we obtained five libraries, and all the samples resulted in having acceptable quality metrics. The only sample having lower mean depth and uniformity parameters (case 5) resulted in a failed Copy Number calculation, which is particularly susceptible, especially to the library uniformity (Table 2).

Consistent with the literature data [10], the samples showed high mutational load (median TMB = 9.5), but no relevant differences from patients with complete response and partial response was observed. The most relevant pathogenic mutations were in PIK3ca, KRAS and PTEN genes, which compared in three sequenced patients out of four, in line with what has been observed on public databases (for e.g., 79% mutated-PTEN, 50% mutated-PIK3ca, 32% mutated-KRAS in endometrial carcinoma CPTAC, Cell 2020; 40% mutated-PIK3ca, 29% mutated-PTEN and 21% KRAS-mutated in endometrial cancer MSK, 2018, cBioPortal). Interestingly, sample #1 resulted in having a mutation with uncertain significance in the CCNE1 gene.

## 5. Discussion

In the last few years, the oncological treatments landscape of solid tumors dramatically changed thanks to the advent of immunotherapy, notably in melanoma, non-small cell lung cancer and kidney cancer, among others; in the gynecological setting, significant responses rates were observed in women with advanced or recurrent EC.

These new drugs showed important improvement in progression-free survival and quality of life, with a more tolerable toxicity profile than chemotherapy. The Garnet trial is a phase I trial treating, with Dostarlimab, patients with dMMR/MSI-H endometrial cancer. It demonstrated a 43.5% (95% CI 34.0% to 53.4%) overall response rate, with 11 complete responses and 36 partial responses, and the median duration of response was not reached. Moreover, Dostarlimab showed a favorable toxicity profile. The majority of treatment-related adverse events were grade 1–2 (75.5%), the most common being fatigue (17.6%), diarrhea (13.8%) and nausea (13.8%). Treatment-related adverse events grade ≥3 occurred in 16.6% of patients, and only 5.5% discontinued treatment due to adverse events [7,11].

Exceptional responses in the metastatic setting showing a “Lazarus effect” are increasing in oncology thanks to new target or immune therapies. Patients with PS ≥ 2 are usually excluded from clinical trials, and thus, almost no data are available on the safety and efficacy of immunotherapy in this population. Nevertheless, Pietrantonio and colleagues demonstrated that patients with PS ECOG ≥ 2 may still benefit from anti-PD(L)1-based regimens and even achieve long-term disease control. Indeed, they report 27 patients with different MSI-H or dMMR tumor types, including one patient with EC, treated with anti-PD-1 with or without anti-CTLA-4 therapy, after failure of at least one prior treatment line, with baseline ECOG PS of 2 (74%) or 3 (26%). In 6 weeks, 52% of patients reached ECOG PS 1 and 30% of patients reached ECOG PS 0 in a median time of 10 weeks [12]. A Lazarus response was also described in three patients affected by advanced non-small cell lung cancer with strong expression of PD-L1 (>50%) with a PS ECOG 4 at the treatment baseline. They were treated with immune checkpoint inhibitors and demonstrated a very rapid improvement of PS ECOG to 1 or 0 [13]; in these cases, the median time to clinical response was 5 weeks (range 1–7 weeks).

In the gynecological oncology setting, Bartoletti et al. reported a case of a patient affected by recurrent dMMR EC with pulmonary and hepatic lesions. Thanks to the molecular profiling of the disease, the patient was treated with Dostarlimab, due to the expanded access program available in Italy, with an excellent radiological response both at the pulmonary and hepatic levels [14]. Time to clinical response was not described; however, he reported a partial response at the first radiological evaluation (11 weeks from treatment’s start), consistent with our experience.

We describe six cases of advanced/recurrent dMMR EC, quickly worsened to a PS ECOG 3 or 4, exclusively due to disease burden, but with excellent and rapid clinical response (median time 6 weeks; Table 1) and radiological partial response in all cases at the first CT-scan evaluation. Our data are in line with what was reported in the literature. Firstly, we report cases of patients, some also terminally ill, with poor PS and critically high disease burden, with rapid improvement of patients’ health conditions during the very first days of treatment. Moreover, to the best of our knowledge, the only case series of the Lazarus effect during ICIs available in the literature [12,13] reported a median time to clinical response of 6 and 5 weeks, respectively. No patients interrupted treatment due to immunotherapy-related adverse events, leading to a good quality of life.

In our case series, two patients also underwent palliative radiotherapy for lytic bone lesions and for a bleeding vaginal lesion. Radiotherapy was performed just before starting immunotherapy treatment. In 1953, Mole described an immune-mediated response to radiation by tumor cells located far from the irradiated site, called the abscopal effect. There is now a growing consensus suggesting that combining radiotherapy with immunotherapy provides an opportunity to boost abscopal response rates [15].

In solid tumors, according to ESMO guidelines, ECOG PS is an important factor used to define the best treatment. Indeed, patients with poor PS (ECOG PS 3–4) are usually not eligible for chemotherapy in light of the potential treatment-related toxicities. Therefore, when it comes to the special population of frail patients, the use of anti-PD-1/PD-L1 agents may be justified by virtue of their favorable toxicity profile and long-term efficacy. The PePS2, CheckMate 171 and CheckMate 153 trials revealed anti-PD-1 to be safe in patients with a PS of 2, and showed no difference in the incidence of irAEs between the PS 2 and PS 0–1 populations [16,17,18].

Immune checkpoint inhibitors led to seismic changes in the treatment of several tumor types. The favorable toxicity profile and the long-term effectiveness of anti-PD-1/PD-L1 agents can justify administration in specific patients’ subgroups; for example, in patients with advanced cancers, reduced life expectancy and poor PS. However, a response takes usually weeks, and this might raise several concerns. Critically ill patients with poor ECOG PS are usually excluded from clinical trials, even though three small real-world series reported the efficacy and safety of anti-PD-1/PD-L1-based therapy in ECOG PS 2 patients with different solid neoplasia [19,20,21]. The potential occurrence of the “Lazarus response” to ICIs poses a great challenge to oncologists. The expected high response rate to treatment, the good toxicity profile of ICIs and the long-lasting survival predicted by MSI-high status might outweigh the questions of treating patients with end-stage MSI-high cancers and poor life expectancy. Even though limited by its retrospective nature and small sample size, our real-world experience showed that it is safe to administer Dostarlimab in critically ill patients with dMMR/MSI-high recurrent and metastatic endometrial cancer.

Finally, no significant copy number alterations/LOH emerged by our analysis on this preliminary series, confirming that, generally, the majority of cancers are distinguished in classes dominated by either mutations or copy number changes.

## 6. Conclusions

MSI status should be performed in all patients with EC as a predictive biomarker to ICIs. Dostarlimab administration is safe and feasible in patients with recurrent dMMR EC, which have poor performance status: immunotherapy is an effective and low-toxicity option for these patients. The best supportive care simultaneous to active oncological treatment in these patients is mandatory to retrieve the best results [10].

## Figures and Tables

**Table 1:** Summary of time to clinical response and best radiological response.

Case N°	Time to Clinical Response (from PS ECOG 3–4 to 0–1)	Radiological Best Response
1	3 weeks	CR
2	3 weeks	PR
3	6 weeks	PR
4	9 weeks	PR
5	6 weeks	CR
6	6 weeks	PR
All	Median time 6 weeks	

ECOG Eastern Cooperative Oncology Group; PS Performance Status; PR Partial Response; CR complete response.

**Table 2:** Summary of patients characteristics’.

Case N°	Age	FIGO Stage at Diagnosis	Histotype	Surgery	N° Previous Therapies	ECOG PS Start Dostarlimab	Best Response	TMB	Status MMR	NGS–Pathogenic Variants or VUS
1	54	IB	Endometrioid	YES	1	4	CR	10.43	deficient	(*p53*wt) *CVR1, PIK3CA, PTEN, KRAS, CCNE1*
2	69	IA	Endometrioid	YES	1	3	PR	2.84	deficient	(*POLE* wt; *p53*wt) *PIK3CA, PTEN, KRAS, TP53, BCOR*
3	59	IB	Endometrioid	YES	1	3	PR	19.84	deficient	(*POLE* wt; *p53*wt) *MTOR, PIK3CA, PTEN, KRAS, DICER1, RNF43*
4	73	IVB	Endometrioid	No	1	3	PR	37.83	deficient	*PIK3CA, PTEN, ARID1A*
5	61	IIIC	Mixed (60% serous + 40% endometrioid)	Yes	2	3	CR	ne	deficient	ne
6	75	II	Endometrioid	Yes	1	3	PR	4.93	deficient	*AKT1*, *FBXW7*

FIGO International Federation of Gynecology and Obstetrics; N° number; ECOG Eastern Cooperative Oncology Group; PS Performance Status; CR complete response; PR partial response; MMR mismatch repair; ne not evaluable; VUS variant of uncertain significance; wt = wild type; NGS = Next Generation Sequencing.

## Data Availability

Not applicable.
